# On the mathematical modeling of wound healing angiogenesis in skin as a reaction-transport process

**DOI:** 10.3389/fphys.2015.00262

**Published:** 2015-09-30

**Authors:** Jennifer A. Flegg, Shakti N. Menon, Philip K. Maini, D. L. Sean McElwain

**Affiliations:** ^1^School of Mathematical Sciences, Monash UniversityMelbourne, VIC, Australia; ^2^The Institute of Mathematical SciencesChennai, India; ^3^Wolfson Centre for Mathematical Biology, Mathematical Institute, University of OxfordOxford, UK; ^4^Institute of Health and Biomedical Innovation and School of Mathematical Sciences, Queensland University of TechnologyBrisbane, QLD, Australia

**Keywords:** wound healing, mathematical modeling, reaction-diffusion systems, mathematical biology, skin wounds

## Abstract

Over the last 30 years, numerous research groups have attempted to provide mathematical descriptions of the skin wound healing process. The development of theoretical models of the interlinked processes that underlie the healing mechanism has yielded considerable insight into aspects of this critical phenomenon that remain difficult to investigate empirically. In particular, the mathematical modeling of angiogenesis, i.e., capillary sprout growth, has offered new paradigms for the understanding of this highly complex and crucial step in the healing pathway. With the recent advances in imaging and cell tracking, the time is now ripe for an appraisal of the utility and importance of mathematical modeling in wound healing angiogenesis research. The purpose of this review is to pedagogically elucidate the conceptual principles that have underpinned the development of mathematical descriptions of wound healing angiogenesis, specifically those that have utilized a continuum reaction-transport framework, and highlight the contribution that such models have made toward the advancement of research in this field. We aim to draw attention to the common assumptions made when developing models of this nature, thereby bringing into focus the advantages and limitations of this approach. A deeper integration of mathematical modeling techniques into the practice of wound healing angiogenesis research promises new perspectives for advancing our knowledge in this area. To this end we detail several open problems related to the understanding of wound healing angiogenesis, and outline how these issues could be addressed through closer cross-disciplinary collaboration.

## Introduction

The process of successful skin wound healing involves highly complex coordinated interactions of many different cell types, tissues and biochemical mediators (Martin, 1997). The skin is comprized of two main layers: the epidermis and dermis. The epidermis is the outer layer that contains no blood vessels and acts as a barrier against water loss and infection. The blood vessels that supply oxygen and nutrients to the skin and remove metabolic waste products are found in the underlying dermis, which is separated from the epidermis by the basement membrane. The successful healing of a full-thickness wound—where both the epidermis and dermis are damaged (Brown, 2005)—is dependent on intrinsic factors, including oxidant species (Yahagi et al., 2000; Sen et al., 2002), lactate (Hunt et al., 1978; Trabold et al., 2003), and hypoxia-inducible factors (Diegelmann and Evans, 2004), as well as extrinsic factors, such as medication (Wicke et al., 2000), emotional stress (Cole-King and Harding, 2001), nutrition (Casey, 1998), and age (Gosain and DiPietro, 2004). A critical step in the wound healing process is angiogenesis: the growth of capillaries from pre-existing vasculature in the undamaged surrounding tissue (as distinct from vasculogenesis: the formation of new blood vessels when a preexisting vasculature does not exist; see Bauer et al., 2005b). Healing cannot proceed smoothly without these newly formed blood vessels, as they supply oxygen—a vital component for successful repair (Knighton et al., 1983; Siddiqui et al., 1997; Tompach et al., 1997; Leach et al., 1998; Babul and Rhodes, 2000; Hunt and Gimbel, 2002; Kalliainen et al., 2003; Tandara and Mustoe, 2004; Gajendrareddy et al., 2005) that controls the activity of cells in the wound (Tompach et al., 1997), and which is needed for continued angiogenesis (Gordillo and Sen, 2003).

Angiogenesis is central to other vital processes, such as embryogenesis and ovulation, as well as to pathologies, including chronic inflammatory disorders and solid tumor growth (Polverini, 1995). In fact, tumor growth and wound healing have further striking similarities, such as changes in cell–cell attachment and rearrangement of the tissue microenvironment. This has led to tumors being described as “wounds that do not heal” (Dvorak, 1986). There are, however, key differences between tumor-induced angiogenesis and wound healing angiogenesis. For instance, angiogenesis associated with wound healing is more tightly regulated (Chaplain and Byrne, 1996), and the newly formed blood vessels regress (reduce to levels in normal tissue) after healing is complete (Polverini, 1995). Nevertheless, the study of solid tumors has indirectly contributed to the understanding of wound healing, for instance by uncovering the fact that the extent of capillary growth is mediated by the half-lives of angiogenic regulators (Wong et al., 1996). In 1996, Chaplain and Byrne notably commented that wound healing and tumor growth can be modeled through similar mathematical approaches (Chaplain and Byrne, 1996). Since then, experimental and theoretical advances in tumor-induced angiogenesis research have contributed to the study of wound healing and *vice versa* (see, for example, Chaplain, 2000).

As *in vivo* investigations are difficult to perform in a non-invasive manner, biologically-realistic mathematical models provide a useful alternative framework for examining wound healing (Cook, 1995). The development of theoretical models that describe the components of wound repair, together with their synergistic or antagonistic interactions, can provide a means to identify elements of the process that can be manipulated in a rational, mechanism-based strategy for improved clinical management. Furthermore, such models can give insight into the relative importance of the interlinked, underlying processes, thus aiding in the enhancement of treatment methodologies (McDougall et al., 2006). It is well-established that such models have the potential to generate theoretical predictions that could not have been anticipated otherwise, thereby stimulating further biomedical research and reducing the need for difficult and costly experiments (Byrne and Owen, 2002).

There have been several reviews of the mathematical models developed to describe angiogenesis in both wound healing (Sherratt and Dallon, 2002; Geris et al., 2010a,b) and solid tumors (Mantzaris et al., 2004; Alarcón et al., 2006a; Chaplain et al., 2006). For instance, Mehidizadeh et al. review models of vascularization—the formation of new blood vessels when a pre-existing vessel does (angiogenesis) or does not (vasculogenesis) exist—in tissue engineering scaffolds, covering much of the literature of both wound healing and tumor-focused angiogenesis (Mehidizadeh et al., 2014), while Spill et al. review mesoscopic and continuum models of angiogenesis, focusing on the tumor-based literature (Spill et al., 2015). However, to our knowledge, there are no published reviews on the seminal continuum models that describe wound healing angiogenesis as a reaction-transport mechanism.

In this pedagogical review, we detail the mathematical principles involved in developing such models, in a way accessible to researchers who are unfamiliar with these techniques. Through this approach, we aim to draw attention to the structural elegance and predictive capabilities of models of this nature, and bring into focus the necessity of interdisciplinary research in this area. While the literature on simple wound healing cell migration assays (Maini et al., 2004a,b) and on tumor spheroids grown *in vitro* (Roose et al., 2003; Loessner et al., 2013) reveal many instances of joint experimental and mathematical work, there is a large body of complex theoretical investigation into numerous aspects of wound healing that has, more or less, evolved with minimal experimental cross-verification. With this review, we hope to illustrate how theoretical models can throw light on important aspects of wound healing angiogenesis, and demonstrate the mutual benefit of closer collaboration between experimentalists and mathematicians on this far-reaching problem. It is beyond the scope of this paper to review in detail all of the published mathematical models that describe wound healing or angiogenesis. We have therefore selected a subset of models that, in our opinion, illustrate the mathematical principles behind developing a reaction-transport model of wound healing angiogenesis and the type of insight that this framework can provide.

This paper is structured as follows: in the next section we describe the biology of wound healing, focusing on the factors that regulate angiogenesis. We then present a detailed overview of the general principles underlying the reaction-transport framework that has commonly been used to model wound healing angiogenesis, and review several key models of this nature. We finish by highlighting four key issues related to the advance of research in this area. Firstly, there are several open questions regarding the way that the evolving vascular network is modeled within a reaction-transport framework, including the processes of anastomosis and branching. Secondly, more work needs to be carried out to provide reliable estimates for the many, often unknown, parameter values in reaction-transport models of wound healing angiogenesis. Next, as reaction-transport models of wound healing angiogenesis have largely been formulated in 1D, it would be fruitful to extend the modeling principles to higher dimensions. Finally, further progress can be made in the areas of discrete, hybrid and multiphase modeling of wound healing angiogenesis by drawing on the existing literature for tumor-induced angiogenesis.

## Biology of wound healing

The nature of the healing pathway depends on the severity of the wound. In full-thickness wounds, successful repair is thought to progress through four stages (Ayello and Cuddigan, 2004): haemostasis, inflammation, proliferation, and remodeling, although these phases are interconnected and overlapping (Enoch et al., 2006). In this review, we restrict our focus to wounds that heal without being surgically sealed (“second intention”), in which healing occurs through the replenishment of granulation tissue and extracellular matrix (Enoch and Leaper, 2005; Kumar and Leaper, 2008). As the dermal wound healing pathway has been detailed in a number of insightful reviews (Singer and Clark, 1999; Enoch and Leaper, 2005; Gurtner et al., 2008), we limit our discussion below to the essential components of this process.

**Haemostasis** is initiated upon injury to the dermal tissue, and typically proceeds over the course of a few hours. As blood from the severed capillaries, carrying platelets, and fibrinogen (Sheffield and Smith, 2002), streams into the wound, the immediate reaction of the body is aimed at impeding blood loss (Molnar, 2007). In response to the exposed epithelium, fibrinogen is activated to form a fibrin mesh that provides a provisional matrix for cell migration, and traps platelets (Enoch and Leaper, 2005) that adhere to the ruptured blood vessels, thereby preventing further blood loss (Sheffield and Smith, 2002). As platelets come into contact with damaged extracellular matrix (ECM) components, they release clotting factors, leading to the formation of a blood clot within the wound site (Diegelmann and Evans, 2004). Platelets within the clot stimulate the subsequent inflammatory response through the release of chemical stimuli such as transforming growth factor β (TGF-β) and vascular endothelial growth factor (VEGF) (Diegelmann and Evans, 2004; Bauer et al., 2005b).

The **inflammation** phase, which typically lasts a couple of days, is characterized by an influx of immune cells (neutrophils and monocytes), which are attracted to the wound site by a diverse range of chemoattractants (Enoch and Leaper, 2005). On arrival, neutrophils phagocytose foreign particles, bacteria, and the blood clot (Mathieu, 2002), while releasing pro-inflammatory cytokines (Hübner et al., 1996) that stimulate the invasion of fibroblasts from the surrounding undamaged ECM (Martin, 1997). Subsequently, monocytes change their phenotype to form macrophages, which actively migrate up the chemoattractant gradient, while consuming the necrotic material in their path, including dead neutrophils (Bellingan et al., 1996), and releasing growth factors such as macrophage-derived growth factors (MDGFs), endothelial growth factors (EGFs), VEGF and TGF-β (Tandara and Mustoe, 2004). An insufficient supply of macrophages can impede angiogenesis and other subsequent components of the healing process (Enoch and Leaper, 2005).

The fibroblast is the dominant cell during the **proliferation** phase of healing (Bauer et al., 2005b). Its survival and activity is crucially dependent on the presence of sufficient oxygen (Gordillo and Sen, 2003). Fibroblasts produce collagen, a major component of the ECM (Enoch et al., 2006). This provides a scaffolding upon which the vascular network can extend into the wound space (Sheffield and Smith, 2002). During this phase, keratinocytes, on being activated by growth factors, migrate and proliferate to create an epithelial layer that seals the wound (Sheffield and Smith, 2002). Growth factors also stimulate the release of proteases from the endothelial cells of vessels in neighboring healthy tissue (Mantzaris et al., 2004), as well as from keratinocytes, fibroblasts, and macrophages (Trengove et al., 1999). These proteases digest the basement membrane that separates blood vessels from surrounding connective tissue, allowing endothelial cells from neighboring blood vessels to escape the confines of their parent vessel (Bauer et al., 2005b). Growth factors such as VEGF and TGF-β stimulate the systematic rearrangement of these cells (Diegelmann and Evans, 2004), which elongate and align to form capillary sprouts (Mantzaris et al., 2004) that extend away from the original vessel (Pettet et al., 1996a), signaling the start of angiogenesis (Gordillo and Sen, 2003). Angiogenesis is highly regulated through the activity of growth factors, cytokines and inhibitors (Crowther et al., 2001), and aids in the transport of neutrophils and macrophages into the wound bed (Crowther et al., 2001). During this stage, sprout extension is facilitated by the migration of endothelial cells toward the chemical attractant, and their continued proliferation (Diegelmann and Evans, 2004). Capillary tips and sprouts join to form a network of new blood vessels, which subsequently supply the wound with oxygen, thereby ameliorating tissue ischemia and hypoxia (Crowther et al., 2001), as well as nutrients necessary for facilitating further healing (Clark, 1996). The oxygen levels in the tissue play a crucial role during this stage; although mild hypoxia is known to act as a trigger for angiogenesis, extreme hypoxia can severely inhibit it (Sen et al., 2009). As healing progresses, a structural “wound healing unit” of macrophages, fibroblasts, ECM and capillary sprouts migrates through the wound site (Arnold and West, 1991; Tompach et al., 1997). Once blood vessels have established a network over the entire wound space, the oxygen levels are returned to normal (Diegelmann and Evans, 2004).

While the proliferative stage of healing typically lasts several weeks (Brown, 2005), the subsequent **remodeling** phase lasts for several months or even years (Sheffield and Smith, 2002). During this period, fibroblasts replace the provisional fibrin mesh with a collagen matrix (Calvin, 1998) that they subsequently remodel and reorganize. The remodeling phase includes wound contraction, during which fibroblasts, upon receiving chemical and mechanical cues, differentiate into myofibroblasts that align themselves along the newly formed ECM and generate tensile strength across the wound (Bauer et al., 2005a). Moreover, as the tissue is no longer hypoxic, there is a marked decline in vascular density and an increase in cellular apoptosis (Lokmic et al., 2006). Typically, complete wound contraction occurs during this phase and the wound tensile strength increases to around 80% of normal within a span of 2 years (Natarajan et al., 2000).

Disruptions to one or more of the stages of healing can lead to serious pathologies such as hypertrophic scars (Ghahary and Ghaffari, 2007), keloid scars (Funayama et al., 2003), and non-healing wounds (Thackham et al., 2008). Hypertrophic and keloid scars involve an overstimulated healing response in the production of collagen during the proliferative phase of healing, thought to be a result of altered keratinocyte-fibroblast interactions (Funayama et al., 2003; Ghahary and Ghaffari, 2007). Non-healing, or chronic, wounds—also known as ulcers (Hermans, 2010)—are characterized by a failure of the repair process to re-establish functional integrity in the expected time frame (Lazarus et al., 1994). A chronic wound is often a surface manifestation of an underlying issue, such as arterial disease or diabetes, and treatment typically depends on the wound etiology. For example, diabetic ulcers are commonly treated with debridement of the wound tissue, ulcers caused by arterial deficiency are treated by restoring arterial inflow (using, for example, a stent) and venous leg ulcers are treated with compression bandages (Thackham et al., 2008). Extreme wound hypoxia is a common cause of the dysfunction of the healing process (Sen et al., 2009) and prolonged hypoxia is considered one of the most common causes of chronic wounds (Mathieu, 2002; Thackham et al., 2008). Roy et al. describe healing in ischemic wounds—where there is a restriction of blood supply to the tissue, causing hypoxia—in contrast to non-ischemic (normal) wounds (see Figure 3A in Roy et al., 2009). These data are from porcine (pig) wounds—a commonly used experimental model of healing in human wounds (Sullivan et al., 2001). Chronic wounds have in recent times been treated with oxygen therapy designed to restore oxygen levels (Thackham et al., 2008), with the application of bioengineered skin equivalents that provide an artificial ECM to promote the proliferation and migration of cells (Harding et al., 2002) and exogenous growth factors that stimulate cellular proliferation and migration (Upton et al., 2011).

In recent decades, healthcare systems worldwide have struggled to deal with the rising costs associated with chronic, non-healing, skin wounds. In the United States alone, the treatment of chronic wounds has been estimated to cost $25 billion annually (Sen et al., 2009). Those suffering from chronic wounds experience significant pain, reduced mobility and a general decrease in quality of life (Chase et al., 2000). Moreover, wounds of this type can persist for many years (Cullum et al., 2004), often leaving no option but to amputate the limb (Lerman et al., 2003). The prevalence of leg ulcers increases with age, with those aged over 60 most at risk (Kucharzewski et al., 2003), and with the expected increase in the aged population, associated treatment costs are forecast to rise (Diegelmann and Evans, 2004). It is therefore crucially important to understand the factors that lead to the dysregulation of wound healing in order to develop improved treatment strategies.

While biological experiments and clinical trials have thus far facilitated much of the current understanding of wound healing, there are many aspects of this process that are difficult, or even impossible, to investigate given current experimental techniques. To that end, theoretical descriptions offer a way to explain and potentially predict certain wound healing behaviors.

## Modeling wound healing angiogenesis as a reaction-transport process: a pedagogical overview

When attempting to describe aspects of biological phenomena in a mathematical model, the broadest guiding principle is that the model should only be as complicated as is warranted by the underlying research question and/or the resolution of experimental data available. While all models of wound healing angiogenesis are subject to several simplifying assumptions, the exact form of the resulting model may vary considerably. In the following, we present a brief overview of the typical decisions involved in the process of developing mathematical models of wound healing angiogenesis, and focus in particular on the use of the reaction-transport framework as a description of the interactions between the constituent species.

### Wound domain (geometrical considerations)

Arguably, the first decision relates to the spatial scale under consideration, i.e., whether one intends to describe behavior at the level of cells, tissues, or across multiple scales. In this review, we focus on tissue-scale models of the wound domain, which are the most commonly used theoretical framework for studying wound healing angiogenesis. As the shape of the wound can impact the timescale and other aspects of the healing process, another important decision relates to how the wound geometry can be approximated, i.e., whether to assume that the wound is roughly circular, rectangular or irregular. Moreover, depending on the nature of the wound under consideration, a decision is made whether to model this process in 1, 2, or 3 spatial dimensions. A common assumption in models of wound healing is that the wound is much longer than it is wide or deep, so that only one spatial variable, *x*, needs to be considered. In such one-dimensional (1D) models, healing occurs from the wound edges (at *x* = *L* and *x* = −*L*) toward the wound center (*x* = 0), with healing from the bottom neglected. This model could be simplified further by assuming symmetry around *x* = 0 and hence simply describing the behavior in the region 0 < *x* < *L*. Although comparatively less realistic than analogous higher dimensional models, 1D models offer conceptual simplicity and are potentially analytically tractable. Hence, a 1D geometry (Cartesian or polar coordinates) has been commonly adopted in models of wound healing angiogenesis (Pettet et al., 1996a,b; Olsen et al., 1997; Byrne et al., 2000; Gaffney et al., 2002; Maggelakis, 2003, 2004; Schugart et al., 2008; Xue et al., 2009; Flegg et al., 2012a).

**Figure 1:**
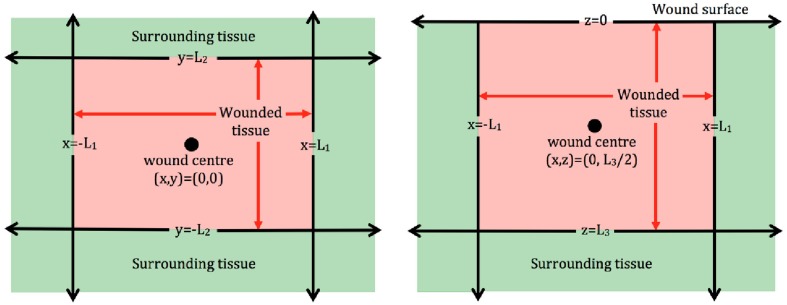
**Schematic of 2D wound domains. Left** : Plan view of a rectangular wound that is parallel to the skin surface. Here *x* = −*L*_1_, *x* = *L*_1_, *y* = −*L*_2_, and *y* = *L*_2_ represent the four wound edges. **Right**: Side view of a rectangular wound that is perpendicular to the skin surface. Here *x* = −*L*_1_ and *x* = *L*_1_ represent the wound extent parallel to the surface, which is located at *z* = 0, and the wound depth is *z* = *L*_3_.

In order to examine the role that the wound shape or surface extent plays in the healing process, two dimensional (2D) models are often employed. Models of this type could be used to describe wounds with a comparatively larger surface extent, for instance burn wounds, and provide a bird's eye view of the wound surface (Figure 1, left subplot). Examples of 2D models of wound healing angiogenesis include Machado et al., 2011 and Valero et al., 2012. Alternatively, 2D models may be used to describe angiogenesis in healing wounds that extend deep into the dermis, in which case they provide a cross section of wound depth vs. length (Figure 1, right subplot), as in the approaches adopted in Olsen et al. (1997) and Vermolen and Javierre (2011).

For wound domains similar to those in Figure 1, a Cartesian coordinate representation is appropriate, whereas in the case of circular wounds, a polar coordinate system is typically adopted in order to take advantage of the symmetry inherent to such wounds. In the following, we focus on models cast in a Cartesian coordinate system, although the modeling principles outlined below extend naturally to other coordinate systems.

### Modeling framework

Subsequent to adopting a choice of wound geometry, an assumption needs to made about the expected number of chemical molecules (or number of cells) per unit volume in the wound. If this value is small, the wound may be better described using a discrete model, in which the stochastic dynamics of individual particles are considered. Otherwise, it would be more appropriate to use a continuum model that describes the net evolution of the chemical concentration or cell densities. The choice of modeling approach (discrete vs. continuum) is also affected by factors such as the nature of comparable experimental data (i.e., whether it contains information related to individual cells or to macroscopic tissue behavior) and the desired level of spatial resolution that is required to answer the research question. Recently developed hybrid simulation techniques can allow cells to be modeled discretely in certain locations in space and time and continuously in others, with the caveat that solutions must be matched across the interface (Flekkøy et al., 2001; Flegg et al., 2012b, 2015; Franz et al., 2013). In general, discrete models can provide a level of detail that continuum models cannot, including being able to specify properties of individual cells. However, as continuum models readily lend themselves to an array of analytical techniques for further study, the majority of wound healing mathematical models to date have been formulated using a continuum approach. Consequently, in this review, we mainly focus on continuum models. Some recently developed discrete modeling techniques are nevertheless briefly discussed in the next section.

### Species to be included in the model

A further key decision relates to the number of interacting species that are needed to adequately describe the process under consideration. This number can vary substantially, depending on the scope of the model. While at least two species are required to describe this process (a minimal model is Gaffney et al., 2002, in which only blood vessels and endothelial cells are considered), there are numerous species that could be considered, including oxygen, inflammatory cells, VEGF, TGF-β, fibroblasts, ECM, etc. Most often, however, a small number of species will suffice, as a model with a large number of species may be difficult to simulate, almost surely impossible to parameterize, and, more importantly, may contain redundant species. The redundancy of a species can be inferred from a simple question: If this species were to be ignored, is it possible to develop a model that displays similar qualitative and/or quantitative behavior? On a more fundamental level, this aspect of model development relates to the identification of those species—and *only* those species—that contribute substantially to the biological process under consideration.

### Development of model equations

As the macroscopic behavior of cells and chemicals can be captured by considering a reaction-transport mechanism, it is a natural framework with which to describe aspects of the wound healing angiogenesis process, such as the propagation of a structural “wound healing unit” of macrophages, fibroblasts, ECM, and capillary sprouts through the wound. The reaction-transport mechanism is outlined in words in Equation (1) and mathematically in Equation (2). If a wound is assumed to contain *N* interacting species, the changes of the concentration (or density) of these species over time and space can be typically described by a corresponding set of *N* partial differential equations (PDEs), using a reaction-transport framework. Put simply, a reaction-transport system is one in which the spatial and temporal changes of one or more substances are influenced by local reactions (mass transformation from one substance to another) and transport (spreading out over space, either randomly or in a directed fashion). Considering a small representative volume, *V*, of the wound, it can be seen that the number of molecules (or cells) can change due to: (i) the net flow of material into this volume and (ii) production/destruction of material inside the volume. This concept of mass conservation can be expressed as:

(1) $$\begin{array}{l} \left\{\begin{array}{l} \text{rate of change of}\\ \text{ species in V} \end{array}\right\}\;=\left\{\begin{array}{l} \text{ net flux of species}\\ \text{through boundaries of V} \end{array}\right\}\\ \;+\left\{\begin{array}{l} \text{net production rate}\\ \text{of species within V} \end{array}\right\} \end{array}$$

and represented mathematically as the set of reaction-transport PDEs:

(2) $$\begin{array}{l} \underset{\text{rate of change of species}}{\underset{︸}{\frac{\partial c_{i}}{\partial t}}}=\underset{\text{Diffusive terms}}{\underset{︸}{\nabla \;\cdot \;\left(D_{i}\nabla c_{i}\right)}}-\underset{\text{advective terms}}{\underset{︸}{\nabla \cdot \left(c_{i}v_{i}\right)}}\\ \;+\underset{\text{source/sink terms}}{\underset{︸}{S(c_{1},\ldots ,c_{N})}} \end{array}$$

where *c*_*i*_(**x**, *t*) represents the concentration (or density) of the *i*th species (*i* = 1, …, *N*) measured in mass per unit volume at time *t* and spatial location **x** (which is expressed in the coordinate system of choice). Here, **D_i_** and **v_i_** represent the diffusivity and the advective velocity of the *i*th species, respectively. The right hand side of Equation (2) is essentially ∇ · (−**F**_*i*_) + *S*, where **F**_*i*_ = −**D_i_**∇*c*_*i*_ + *c*_*i*_**v_i_** is the total flow associated with the *i*th species and *S* contains the source/sink terms (related to net cell or chemical production). The divergence of **F**_*i*_ gives rise to two terms that represent, respectively, the rate of change of *c*_*i*_(**x**, *t*) due to diffusive and advective flow.

When describing chemical species, such as oxygen and chemoattractants, it will usually suffice to consider diffusion as the sole flow term in Equation (2). For cellular species, this diffusive term is often used to model random motion. As cells are typically several orders of magnitude larger than chemical molecules, the random motion of cells is often small compared to the diffusion of chemical species. Furthermore, nonlinear random motion terms are typically used to reflect the observation that cells move into the wound space as a distinct cell front. Sharp-fronted solutions of this nature can be mathematically described by considering a diffusion coefficient that is a non-constant function of the dependent variable (Simpson et al., 2006). While cell random motion can, in principle, be assumed to be anisotropic (directionally dependent), in models of wound healing it is usually assumed that the given species will move randomly at the same rate in all directions. Cook developed models for dermal wound contraction in which anisotropic random motion was used to model the movement of cells in response to an orientated strain environment (Cook, 1995). Advective flow terms have been used to describe the directed motion of cells (e.g., fibroblasts, macrophages, and endothelial cells) during wound healing angiogenesis, including cell motion toward higher levels of substrate (haptotaxis) (Olsen et al., 1997) and chemoattractants (chemotaxis) (Pettet et al., 1996a,b; Flegg et al., 2012a). In this way, **v_i_** in Equation (2) is specified in terms of *c*_*i*_, that is **v_i_** = **v_i_**(*c*_1_, …, *c*_*N*_). In the case where the velocity, **v_i_**, is itself an unknown quantity, an extra equation must be developed to solve the system. As we will discuss, the way cell movement is modeled in the wound space largely determines how angiogenesis is included in a model.

If spatial changes are negligible, i.e., if the system can be considered to be spatially well-mixed, then Equation (2) reduces to a set of temporal ordinary differential equations (ODEs). For example, Bowden et al. recently developed an ODE model of contraction in full thickness diabetic wounds, without angiogenesis (Bowden et al., 2014). However, as angiogenesis involves temporal changes over several weeks, and spatial changes that occur over the wound domain (often of the order of centimeters), continuum models of wound healing angiogenesis have typically preferred the use of PDEs to model spatio-temporal changes.

The source (reaction) terms in Equation (2) model the conversion of mass from one species to another, incorporating processes such as synthesis of chemicals by cells, consumption of oxygen by cells, cell death, chemical decay, and regulation of cell proliferation by chemicals. For example, one way to model anastomosis between a blood vessel and a capillary tip, is to incorporate a loss term in the equation governing the capillary tip (proportional to the number of blood vessels) in order to represent the loss of capillary tips from the system. There are open modeling questions centered around anastomoses, including: how does one capillary tip locate a sprout/tip? Is this a random process, or are they attracted to each other? We will discuss these and other related questions in a subsequent section.

### Initial and boundary conditions

To close Equation (2), knowledge of the initial state of *c*_*i*_(**x**, *t*), as well as its values at the wound boundaries, is required. The initial values could either be informed from available experimental data, or reasonable mathematical assumptions can be imposed. For example, when healing starts, it could be assumed that the wound contains no blood vessels, cells or chemicals; only a fibrin clot. The exact number of boundary conditions for *c*_*i*_(**x**, *t*) depends on the form of the governing equation in Equation (2). In general, for **D_i_** ≠ **0**, boundary conditions are required at the two wound edges for models that use 1D Cartesian coordinates and on the four edges in models that use 2D Cartesian coordinates (Figure 1). The form of the boundary conditions depends on the species in question. It would be natural, for example, to assume that the oxygen concentration at the wound edge is the same level as that in healthy tissue. In certain situations, it may be more appropriate to place a condition on the flow of a species at a boundary, for instance to assume that the flow of a chemical species at the wound edge is proportional to the density of blood vessels.

### Estimation of model parameter values

Continuum PDE models of wound healing angiogenesis often contain a number of parameters that need to be estimated from available experimental data, or taken from the literature. Moreover, some of the model parameters may be inherently difficult to estimate due to the complicated nature of the healing process *in vivo*, in which case an educated guess in the context of the problem must suffice. Nevertheless, in all cases, it is important to test the robustness of the system with respect to changes in these parameters, i.e., to determine whether similar results can be obtained even if the chosen parameter set is subject to perturbations. This parameter sensitivity analysis can help identify those parameters, if any, whose variation may cause large changes in the solution. Obtaining reliable and accurate estimates of the parameters in wound healing models is an open problem that needs more research. In the absence of this, the predictive capacity of these models cannot be fully realized.

### Approaches to solving the equations

Once the governing equations, initial and boundary conditions, and parameter values have been specified, the model takes the form of an initial boundary value problem (IBVP). The resulting system of equations can be solved using either analytical or numerical techniques, although explicit analytical solutions are typically only available for simple models. Very rarely are the equations amenable to rigorous existence-uniqueness analysis. In certain cases, analytical solutions can be obtained for more complex models if approximations are made based on the relative size of parameter values and/or processes; for example, by assuming certain sizes of model parameter values, Pettet et al. derive an expression for the healing wavespeed Pettet et al. (1996b). Most often, due to the nonlinear, coupled nature of the IBVP, the full model is solved using a numerical technique (for a given set of parameter values), so that the change in the species over time and space can be visualized. Several methods exist for solving IBVPs, including finite difference, finite volume and finite element methods. Some existing numerical packages are useful in generating numerical solutions to IBVPs, including MATLAB's pdepe.m (1D) and NAG routines do3pcf.f (1D) and d03raf.f (2D). It is important to note that unless care is taken when choosing and implementing a numerical scheme, some terms in Equation (2) may give rise to numerical errors. For example, equations with advection dominating diffusion can be difficult to simulate with simple numerical schemes and in the absence of diffusion altogether, the governing equation will change from parabolic to hyperbolic. Special techniques to deal with intricate numerical issues may need to be considered (Thackham et al., 2008), and may require part of the numerical code to be written specifically for the system at hand.

## Wound healing angiogenesis: achievements of mathematical modeling

**Figure 2:**
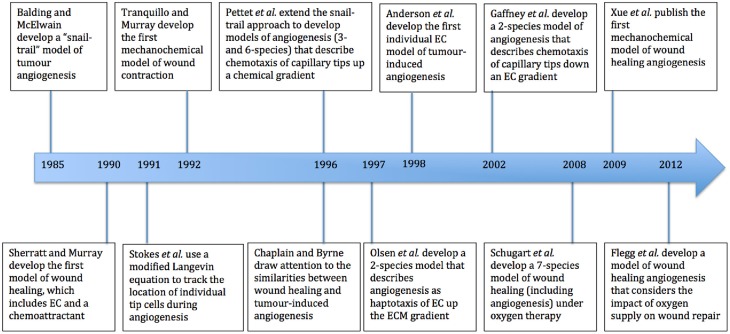
**Timeline of major mathematical models of wound healing and of the process of angiogenesis**. EC refers to endothelial cells and ECM to extracellular matrix.

We now present an overview of a selection of mathematical models that have contributed to the literature on wound healing angiogenesis. These include some that are related to tumor-induced angiogenesis, and others that model the wound healing process without explicitly describing angiogenesis. Figure 2 shows a summary of some of the models discussed in this section, all of which have provided new perspectives on the process of modeling wound healing angiogenesis. While there have been many papers that have made important contributions to this field, it is beyond the scope of this paper to review them all in detail.

In 1985, Balding and McElwain developed the first mathematical model of tumor-induced angiogenesis (Balding and McElwain, 1985) (Figure 2). This model, referred to as the “snail-trail” model, was based upon a model of fungal growth by Edelstein et al. (Edelstein, 1982), and describes the spatiotemporal evolution of 3 species, namely, blood vessel density (*b*), capillary tip density (*n*), and chemoattractant (tumor angiogenesis factor) concentration (*c*) in a 1D tumor, through a set of coupled PDEs. The capillary tip density is governed by the equation:

(3) $$\begin{array}{l} \underset{\text{rate of change of capillary tip density}}{\underset{︸}{\frac{\partial n}{\partial t}}}=\underset{\text{chemotaxis}}{\underset{︸}{-\frac{\partial }{\partial x}\left(n\chi \frac{\partial c}{\partial x}\right)}}+\underset{\text{sprouting}}{\underset{︸}{\mathrm{\alpha c b}}}\\ \;-\underset{\text{anastomosis}}{\underset{︸}{\beta b}} \end{array}$$

where α and β are the rates of sprouting and anastomosis, respectively. Here, the first term on the right hand side of Equation (3) represents the chemotactic response of capillary tips toward higher concentrations of chemoattractant, where tip velocity is given by $v=\chi \frac{\partial c}{\partial x}$ and where χ is a positive constant. The model assumes that as the capillary tips move, they leave blood vessels behind them. That is, if the capillary tips, *n*, move with a velocity, **v**, then the rate of increase (production/extension) of blood vessels is given by $\left(n\text{v}\right)\cdot \hat{\text{v}}=\text{F}\cdot \hat{\text{v}}$, where $\hat{\text{v}}$ is a unit vector in the direction of **v** and **F** is the total flux vector. The blood vessel density in this 1D model is governed by

(4) $$\begin{array}{l} \underset{\text{rate of change of blood vessel density}}{\underset{︸}{\frac{\partial b}{\partial t}}}=\underset{\text{angiogenesis}}{\underset{︸}{-nv}}-\underset{\text{regression}}{\underset{︸}{\gamma b}} \end{array}$$

where γ is the rate of vessel regression (reduction in density to levels in normal tissue), *v* is the tip velocity, and the first term on the right hand side of Equation (4) represents the increase in vessel density due to extension of the capillary tips. To date, this snail-trail model has not been extended to 2D, and this remains an open problem. Nevertheless, the model has proven to be a robust framework upon which a range of subsequent mathematical models of angiogenesis have been developed, including for the specific case of wound healing angiogenesis (see, for example, Pettet et al., 1996a,b; Flegg et al., 2012a).

The first mathematical model of wound healing, proposed by Sherratt and Murray (1990) in the context of epidermal healing, contained just two species: a chemical, *c*(*x, t*), that activates epidermal cells, *n*(*x, t*), to proliferate along a 1D wound. The model did not include angiogenesis, but it is the seminal paper for modeling wound healing and it introduces several ideas that subsequent models have adopted, including how cell motion, death and proliferation can be described mathematically. An early contribution to modeling angiogenesis was the work of Stokes and Lauffenburger, who tracked the location of individual tip cells during angiogenesis (Stokes and Lauffenburger, 1991). This was achieved using a Langevin equation, modified to include a drift term for chemotaxis.

Although seminal and influential, these early models simply considered the chemical and cellular interactions that occur in the wound. An important missing component was the role that stress and mechanical forces play in mediating the healing process. It is in fact fairly straightforward to incorporate such forces into the reaction-transport framework discussed in the previous section. Recall that we previously stated that **v_i_** in Equation (2) is often specified in terms of the other species in the model, that is **v_i_** = **v_i_**(*c*_1_, …, *c*_*N*_). In order to account for the possibility that **v_i_** is dependent on some external factor, that is, if **v_i_** is an unknown quantity to be solved for, an extra set of equations—typically, balance of momentum equations for *c*_*i*_—must now be imposed in order to solve the system. This will involve making constitutive assumptions for how physical quantities, such as stress and strain, are related (see Valero et al., 2014 for a recent review of the material property choices in modeling wound healing in soft biological tissues). This class of wound healing models, in which both mechanical and chemical interactions between cells and chemicals are considered, are referred to as *mechanochemical*.

The first mechanochemical model of wound healing was developed in 1992 by Tranquillo and Murray (1992), based on a model of morphogenesis. Their description included a conservation of mass equation for fibroblasts and ECM, where each of these species is assumed to undergo passive convection with the same velocity, **v**, due to the contraction of the wound, and a balance of momentum is applied to close the system. Although their model did not include angiogenesis, it has formed the basis for much of the later mechanochemical work that does (e.g., Xue et al., 2009, which we discuss later).

A turning point in this field was precipitated by a paper published in 1996 by Chaplain and Byrne (1996), that highlighted how tumor-induced angiogenesis and wound healing angiogenesis can be modeled in similar ways. The notion that wounds and tumors had similar characteristics and processes was not new: Dvorak et al. commented on the similarities (and differences) in their 1986 paper (Dvorak, 1986). However, Chaplain and Byrne were the first to highlight the similarities in the two angiogenesis processes from a mathematical point of view. This observation kick-started a rapid progression in the development of models of wound healing angiogenesis, leaning on the wealth of literature that existed for tumor-induced angiogenesis.

Contemporaneously, Pettet and coworkers developed two models of wound healing angiogenesis: a 6-species model (Pettet et al., 1996a) and a simplified 3-species model (Pettet et al., 1996b). These models have proven to be highly influential and have inspired many contributions to the modeling of angiogenesis in the past 20 years, including those by Byrne et al. (2000), Chaplain et al. and Flegg et al. (2009, 2010, 2012a).

In their 6-species model, Pettet et al. considered the evolution of capillary tips, *n*, capillary sprouts, *b*, fibroblasts, *f*, chemoattractant, *a*, oxygen, *w*, and ECM, ρ in a 1D domain (Pettet et al., 1996a). The wound was assumed to be 1D where *x* = 0 refers to the center of the wound, while *x* = *L* denotes the wound edge. It was assumed that the capillary tips undergo migration due to random motion and chemotaxis, and hence the total flux of tips is $\text{F}=-D_{n}n^{\alpha }\rho \nabla n+\chi_{n}\left(a\right)\rho n\nabla a$, where *D*_*n*_ is the random motility coefficient, α > 0 is a nonlinear random motion parameter and χ_*n*_(*a*) represents the dependency of the chemotactic coefficient on the chemical concentration. In addition, they incorporated the snail-trail mechanism for the rate of increase of blood vessels, $\text{F}\cdot \hat{\text{v}}=\left(-D_{n}n^{\alpha }\rho \nabla n+\chi_{n}\left(a\right)\rho n\nabla a\right)\cdot \hat{\text{v}}$, where $\hat{\text{v}}$ is a unit vector in the direction of **F**. Finally, it was assumed that movement is from right (the wound edge) to left (the wound center) so that $\hat{\text{v}}=-\text{i}$, and the production term for the blood vessels becomes $D_{n}n^{\alpha }\rho \frac{\partial n}{\partial x}-\chi_{n}\left(a\right)\rho n\frac{\partial a}{\partial x}$ in a 1D domain.

**Figure 3:**
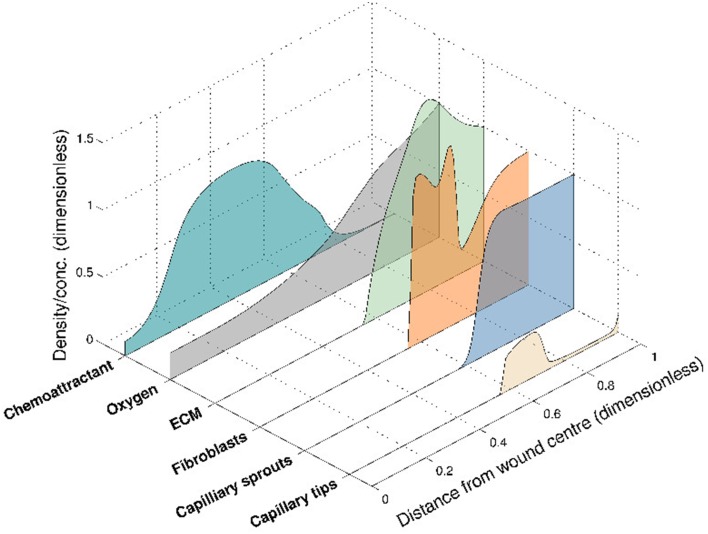
**Numerical simulation of the 6-species Pettet et al. model, showing the spatial distribution of the species within the wound at a certain time for a given set of parameter values**. The wound healing unit moves through the wound space, from wound edge to wound center.

This 6-species angiogenesis model made several important contributions to the literature: many of the important interactions of chemical and cell species were modeled for the first time, including oxygen mediation of chemoattractant production, oxygen-dependent fibroblast proliferation and ECM dependent tip movement. Moreover, clinical insight was gained by numerical simulations that illustrate both healing and stalled wound situations, for distinct sets of parameter values. The model successfully captured the propagation of a wound healing unit through the wound space and an elevated blood vessel density prior to vascular remodeling (Figure 3), both of which are observed experimentally. In this model, chemoattractant is produced in regions where the oxygen concentration is known to promote the release of pro-angiogenic factors (between a lower and upper threshold of the oxygen concentration). The chemoattractant then attracts fibroblasts to migrate into the wound space, laying down ECM as they move. This newly-laid ECM allows capillary tips to migrate further into the wound, toward the high level of chemoattractant. As they move, these capillary tips lay down capillary sprouts according to the snail-trail model. This laying down of sprouts in turn allows more oxygen to be supplied to the wound, which subsequently moves the wound healing unit further into the wound space. As the wound healing unit moves through the wound, the capillary tips behind the wound healing unit are lost due to anastomosis (see Figure 3).

The 3-species model of Pettet et al., implemented the same mechanism as their 6-species model, but used simplified forms for the interactions between capillary tips, blood vessels and a chemoattractant alone. In this 1D model, the capillary tips move in a wound that heals from left (wound edge) to right (wound center), and the rate of increase of blood vessels due to angiogenesis is given by $F=-D_{n}\frac{\partial n}{\partial x}+\chi_{n}n\frac{\partial a}{\partial x}$. The simplified nature of this 3-species model, allowed Pettet et al. to derive a traveling wave representation of the wound healing unit that characterizes the ingrowth of new capillary sprouts that lead to new fully-functioning blood vessels, and thereby establish conditions under which wound healing stalls.

In contrast to most early attempts at modeling wound healing angiogenesis, wherein blood vessel and capillary tip densities were explicitly included, another approach that soon gained favor was to model the endothelial cell (EC) density alone. As ECs make up the lining of blood vessels (and capillary tips), this approach, in effect, models a combination of capillary tip and vessel components. In 1997, Olsen et al. developed a two-species model of wound healing angiogenesis that considers the density of ECs in newly developed capillaries and an ECM substrate (Olsen et al., 1997). The novel contribution of this work is that it focuses not on the directed motion of ECs due to chemical gradients, but rather on how the substrate (ECM) affects both random and directed motion. The random motion of ECs is regulated by the ECM (haptokinesis) and ECs undergo directed motion toward higher densities of ECM (haptotaxis). This approach of modeling the EC density rather than the blood vessel and capillary tip densities had been adopted in tumor-induced angiogenesis modeling in the time prior to Olsen's work (Chaplain, 1995; Chaplain et al., 1995; Orme and Chaplain, 1996, 1997).

Another approach, proposed by Gaffney et al. (2002), is to consider the evolution of both capillary tips, *n*(*x, t*), and capillary sprouts (also called blood vessels and ECs), *b*(*x, t*). As in the models developed by Pettet et al. (1996a,b), the capillary tip density undergoes both random and directed motion. However, the directed motion term is modeled so that tips migrate down the gradient of blood vessels, with $F_{n}\;=\;-D_{1}\frac{\partial n}{\partial x}-D_{2}n\frac{\partial b}{\partial x}$ being the flux in 1D. Gaffney et al. assume that the blood vessels passively follow their leading tip and are hence not capable of independent movement. Hence, the flux of vessels is assumed to be proportional to the flux of the capillary tips: *F*_*b*_ = *k*_5_*F*_*n*_. A detailed traveling wave analysis and asymptotic analysis of the healing unit yields a characterization of the key aspects of the dynamical behavior of the model, including a minimum healing wave speed, maximum capillary tip density and maximum vessel density in terms of model parameters. The analysis leads to predictions about possible ways to increase the level of angiogenic response.

The role of supplemental oxygen in the treatment of wounds was considered in a 7 species model of wound healing angiogenesis by Schugart et al. (2008). Here, the interactions of capillary tips (*n*), capillary sprouts (*b*), fibroblasts (*f*), macrophages (*m*), oxygen (*w*), chemoattractant (*a*), and ECM (ρ) during the healing process were considered. The mechanism of angiogenesis was modeled in a way that extends the work of Pettet et al. (1996a,b) and Gaffney et al. (2002). The flux of the capillary tips was assumed to have a contribution from (linear) random motion and chemotaxis, so that the flux was given by **F_n_** = **v_n_***n* = −*D*_*n*_ ∇ *n* + χ_*n*_(*n*, ρ)∇*a*. The capillary sprouts were assumed to be dragged along by the flux of the tips so that the sprouts move in the same direction, with a modified velocity **v_b_** = *g*_*b*_(*n*)**v_n_**. With a small amount of random motion, Schugart et al. argued that the total flux of capillary sprouts is then $F_{b}=\;-D_{b}\nabla b+v_{b}b=-D_{b}\nabla b-D_{n}\frac{g_{b}(n)}{n}b\nabla n+g_{b}(n)b\chi_{n}(n,\rho )\nabla a$. Numerical simulations of their 7-species model were used to show that (i) extremely hypoxic wound environments cannot sustain vascular growth, (ii) the use of intermittent oxygen may stimulate the angiogenic response, (iii) hyperoxia promotes wound healing but high levels of oxygen can cause healing arrest, and (iv) there is an optimal level of hyperoxia beyond which the beneficial effects of hyperoxia may be reversed.

The wound healing angiogenesis model of Schugart et al. was subsequently adapted by Xue et al. (2009) to include mechanochemical effects. Extending the Tranquillo and Murray mechanochemical model of wound contraction, Xue et al. treat the ECM as a viscoelastic material that moves with a velocity, **v**, which is determined by a momentum balance. This model, which was formulated for a circular wound with a moving boundary, included an additional ODE for the changing position of the wound boundary over time. Numerical simulations of the Xue et al. model demonstrated that impaired macrophage recruitment to the wound site due to insufficient blood supply may impair healing of chronic wounds. It should be noted that other authors had developed mechanochemical mathematical models of angiogenesis and vasculogenesis (not in a wound healing context) prior to this (see for example Manoussaki, 2003 and Tosin et al., 2006).

A recent attempt at synthesizing several of the previous approaches to modeling wound healing angiogenesis was the model by Flegg et al. (2012a). This 1D model described the interactions between capillary tips, *n*(*x, t*), capillary sprouts, *b*(*x, t*), and oxygen concentration, *w*(*x, t*). Flegg et al. made a modeling assumption (consistent with biology) that capillary tips are not capable of random motion, but undergo directed motion down the local oxygen gradient. The flux of tips in 1D was given by $F_{n}=\chi n\frac{\partial w}{\partial x}$. In line with the snail-trail model of angiogenesis, Flegg et al. include a net production term in the capillary sprout density of $-\chi n\frac{\partial w}{\partial x}$. Using asymptotic techniques, this model was used to determine simple criteria for when successful healing can be initiated through the growth of new blood vessels. It was found that healing fails due to the lack or excess of oxygen. Regions of parameter space where healing is either unsuccessful or successful are predicted based on the rate of oxygen consumption (*k*_2_) and oxygen supply (*k*_4_). If a wound has stalled due to a lack of oxygen, it should be possible to initiate healing by a sufficient increase or decrease of the rates of oxygen supply and oxygen consumption, respectively. Using these results, predictions were made of the efficacy of chronic wound treatments, debridement and revascularization surgery.

While the continuum approaches described above have significantly advanced the understanding of wound healing angiogenesis, other modeling approaches have also helped throw considerable light on this process. For example, early work by Stokes et al. (Stokes and Lauffenburger, 1991) used stochastic ODEs to track the location of individual tip cells during angiogenesis. There were also several groups who simulated individual vessels by considering a random walk for ECs (where the continuum limit of the random walk returns an appropriate governing PDE), such as Anderson et al. (Anderson and Chaplain, 1998), Levine et al. (2001a,b), Plank et al. (Plank and Sleeman, 2003), and Kevrekidis et al. (2005, 2006). In this respect, Anderson et al. were the first to model the behavior of individual ECs during angiogenesis, albeit tumor-induced (Anderson and Chaplain, 1998). Machado et al. later modeled wound healing angiogenesis by using random walks to describe the movement of ECs (Machado et al., 2011).

The processes of branching and anastomosis during tumor-induced angiogenesis were described by Bauer et al. through the use of a discrete modeling framework (Bauer et al., 2007). In this model, which is an application of the cellular Potts model, cellular dynamics are characterized by decisions that minimize the total energy in the system and angiogenesis was modeled using a chemotaxis term in the energy equation that encourages cell movement in the direction of increasing chemoattractant concentration. Another approach was that of Bentley et al., who have developed discrete, agent-based simulations of how angiogenic sprouting is mediated by Notch signaling, where ECs have distinct phenotypes: leading tip cells and stalk cells that follow (see, for example, Bentley et al., 2009). As discrete cell-based approaches have already yielded considerable insight into the process of tumor-induced angiogenesis, there is considerable potential for using such techniques to develop improved descriptions of wound healing angiogenesis, and more work needs to be done in this regard.

## Open problems

In this section, we provide an overview of some outstanding problems in wound healing angiogenesis research, and outline the issues (mathematical and/or laboratory-related) that need to be addressed in order to make progress. We focus, in particular, on five open problems, namely, (i) developing more accurate representations of the behaviors of capillary tip and vessel densities, in the contexts of anastomosis, budding and vessel remodeling/maturation, (ii) improving techniques for estimating the (often numerous) model parameters, (iii) extending pre-existing models of wound healing angiogenesis to higher dimensions, (iv) incorporating techniques used in, and ideas gleaned from, the mathematical modeling of tumor-induced angiogenesis, and (v) comparison with experimental data.

### Modeling of the capillary tip and vessel density

#### Anastomosis

**Figure 4:**
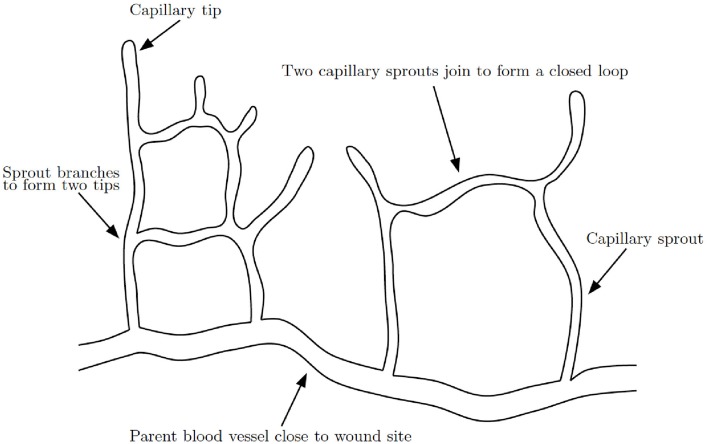
**Schematic of capillary network formation**. Sprouts branch and join to form a closed network of capillaries, modified from Gaffney et al. (2002).

The formation of a capillary network in a healing wound occurs by capillary tip extension from a parent vessel, maturation of capillary tips into capillary sprouts, anastomosis of sprouts to sprouts and sprouts to tips and further branching (Figure 4). The process of anastomosis in wound healing angiogenesis reaction-transport models is typically modeled with very simple terms, such as λ*nb* for when a capillary tip (*n*) meets a vessel (*b*) or λ*n*^2^ when two tips meet, where λ is a positive constant. This approach is overly simplified as it does not include any mechanism by which the two tips, or the tip and vessel, seek each other out and eventually meet. It is difficult to generate new insights on anastomosis from such a model. A complication in extending current models to higher dimensions is that, while a capillary tip and vessel will almost always meet in 2D, in 3D they will almost never meet.

By taking these factors into account when modeling the mechanism of anastomosis, we could gain insight into many questions regarding this complex process. For instance:

- Does a capillary tip find another tip or vessel by moving around randomly? Or are the tips attracted in a more directed fashion? If so, is this process governed by a regulatory chemical?
- Is a tip biased toward other tips rather than vessels? If not, why aren't all the tips lost due to anastomosis early in the healing process? Is this prevented by a requirement that there needs to be some minimal vessel length for anastomosis to occur?

Another extension to consider is modeling the artery and venous connections separately, as the oxygen concentration will be lower in the blood returning to the heart from the extremities (e.g., in the veins).

#### Budding

In the typical reaction-transport model of wound healing angiogenesis (see for example, Pettet et al., 1996a; Schugart et al., 2008), new capillary tips (*n*) can develop from existing vessels (*b*), regulated by a chemical (*a*), modeled by a term such as λ*ab*. The budding of new capillaries from existing ones is known to be tightly spatially regulated (Asahara et al., 1998; Addison-Smith et al., 2008) and this needs to be taken into consideration in mathematical models of wound healing angiogenesis, especially in 2D or higher dimensions.

#### Vessel remodeling/maturation

When the healing unit progresses through the wound space, vessels are quickly produced in response to tip migration, leading to a higher than normal density of vessels behind the moving healing front. After this rapid formation of blood vessels during the inflammatory and proliferative stages of healing, the vasculature system is remodeled/regressed (Lokmic et al., 2006). The standard way to model this is with a logistic term λ*b*(*b*_0_ − *b*). This is insufficient as it does not answer outstanding questions about the vessel remodeling and maturing processes of the vessels. These include:

- What stimulates the removal of the vessels? One possibility is that vessel regression occurs due to reduced production of VEGF and increased levels of oxygen. Would such an assumption be consistent with the interaction of VEGF, oxygen and vessels in normal tissue?
- What determines the vessel density in normal tissue?

Another possibility is that excess vessels are lost because of a physical space limitation (e.g., overcrowding). This could be investigated using a multiphase mathematical model where the volume fraction of vessels and cells are considered. Pries et al. developed a model where the diameter of vessels adapted in response to four local stimuli, namely, endothelial wall shear stress, intravascular pressure, flow-dependent metabolic stimulus, and stimulus conducted along the vascular wall (Pries et al., 1998). This framework has since been used to model the structural adaptation and pruning seen during angiogenesis (Secomb et al., 2013).

The maturing of vessels that remain after regression is an important feature of angiogenesis that should be included in mathematical models of wound healing angiogenesis. Experimental data on the timescale of vessel regression after the healing front has established a network of vessels and how the remaining vessels mature would be informative to future mathematical models.

### Estimating the (many) model parameters

The reaction-transport models of wound healing angiogenesis reviewed in this paper typically have a large (10–40) number of model parameters. While some model parameters could, in principle, be estimated using existing experimental results, this is limited by the following constraints:

- The parameter estimates are typically done in isolation, with a single experimental result informing a single parameter (that is, the parameters in the model are not estimated jointly).
- They are estimated under different laboratory conditions, such as temperature, cell lines and animal model, the effect of which is ignored.
- Experimental uncertainly in the estimation of parameters can often be quantified but is ignored for modeling purposes as there are usually other model parameters that are completely unknown.

Some parameters will be inherently harder to estimate than others. For example, the concentration of oxygen expected in normal tissue will be able to be quantified, however for other parameters, such as those in a nonlinear chemotaxis coefficient, it will be difficult (if not impossible) to untangle their estimation from experimental data. The question is: Could it be possible to design a single (or small number of) experiment(s) whose resulting dataset(s) could be used to estimate the parameters of a complicated model of wound healing angiogenesis? In this case, one could envisage that it would be possible to use a statistical procedure to jointly fit the model parameters. However, it is important to note that even if such a dataset did exist, there are significant potential issues to overcome in order to fit model parameters from governing ODEs and PDEs, including identifiability.

### Extension to higher dimensions

The majority of existing models of wound healing angiogenesis have been formulated in 1D. While this can shed some light on the angiogenesis process, moving forward there is a need to develop models in higher dimensions. There have been preliminary attempts to model wound healing angiogenesis in 2D. However these tend to describe only the evolution of the density of EC or capillary density in isolation (Valero et al., 2012), rather than the laying down of new blood vessels behind moving capillary tips. The “snail-trail” continuum model of angiogenesis developed by Balding and McElwain (1985), and later modified by Gaffney (Gaffney et al., 2002) and Schugart (Schugart et al., 2008) have only been developed and solved in a 1D context (Xue et al. studied a circular wound with assumed radial symmetry Xue et al., 2009). How these models of angiogenesis extend to 2D (and higher) needs consideration. There are existing 2D mathematical models of the wound healing process (without angiogenesis); for example, Menon et al. developed a 2D model of fibroblast-keratinocyte crosstalk during normal and abnormal wound healing (Menon et al., 2012).

Mathematical models of wound healing angiogenesis in higher dimensions will allow detailed investigations of how the development of new blood vessels drives wound closure on realistic wound geometries. For example, Ben Amar et al. developed a model (without angiogenesis) for the advancing epithelium in an initially circular wound and showed that the circular geometry was not maintained during healing, in agreement with experimental observations (Ben Amar and Wu, 2014). In similar work by Ben Amar and colleagues, the moving front of cells in melanoma development was modeled, whereby oxygen was supplied from under the melanoma (e.g., from the dermis) (Balois and Ben Amar, 2014). A mathematical model of the healing front in realistic 2D wound geometries that combines the supply of oxygen from the dermis and the atmosphere above, with a model of oxygen supplied through angiogenesis would be an interesting extension.

### Utilizing advances in tumor-induced angiogenesis modeling

**Figure 5:**
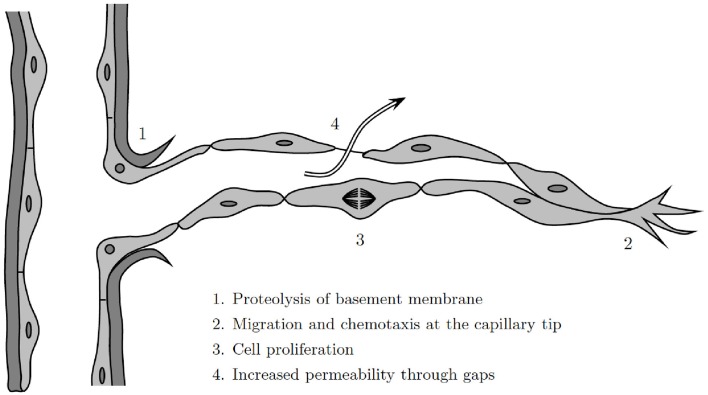
**Illustration of the angiogenesis process at a discrete cell level, modified from Cotran et al. (1999)**.

Historically, progress in the mathematical modeling of wound healing angiogenesis has been made by drawing on work done in the mathematical modeling of tumor-induced angiogenesis. There remain numerous insights and techniques from the tumor literature that could be utilized to develop better models of wound healing angiogenesis. To date, while there has only been a single model of wound healing angiogenesis that treats cells as discrete, i.e., Machado et al., 2011 where ECs are governed by a random walk, there have been a variety of models have been published on multi-scale models of tumor angiogenesis, investigating important aspects of vessel rheology, diameter, and adaption (Alarcón et al., 2005, 2006b; Owen et al., 2009). Indeed, the decisions of individual cells play an important role in wound healing angiogenesis: the basement membrane that surrounds the blood vessels must be degraded to allow ECs to migrate through the walls of the parent vessel (Figure 5).

### Comparison with experimental data

Models of wound healing angiogenesis should be developed in close collaboration with clinicians and/or biologists to ensure the models capture the biology accurately enough to answer the research question, and so that the model predictions can be compared directly with experimental data being collected. Once the model parameter values are fitted and/or estimated (see open problem discussed above on estimating model parameters) it will be important to compare the predictive ability of the models against available experimental data. For instance, with the increased use of digital tracing systems, such as Smith & Nephew's Visitrak™ system, data on wound surface area and geometry, as well as wound depth (Schultz et al., 2005) during the healing process, are becoming available. Furthermore, model predictions can be compared to imaging of *in-vitro* experiments on wound cell behavior (e.g., proliferation, death and migration). For example, Machado et al. compared their theoretical model of angiogenesis in 2D to confocal microscopy images of perfused vascular segments within a mouse wound (Machado et al., 2011).

## Discussion

Over the last 30 years, considerable effort has gone into developing mathematical models of the wound healing process. The use of a reaction-transport framework to model wound healing angiogenesis has helped throw considerable light on the interactions between the constituent species of this critical component of the wound healing pathway. Through this review, we hope to place in context the large body of reaction-transport models of wound healing angiogenesis. In order to highlight the elegance and utility of such models, we detail the conceptual principles underlying model development, including the simplifying assumptions that need to be made, and draw attention to some key models that utilize this approach, focusing in particular on 1D models (Pettet et al., 1996a,b; Gaffney et al., 2002; Schugart et al., 2008; Flegg et al., 2012a).

Although there have been numerous successful attempts at mathematically describing angiogenesis in the context of vascular tumor growth, many of these modeling principles, in particular discrete modeling frameworks for cellular species (Bauer et al., 2007; Drasdo and Hohme, 2005; Owen et al., 2009; Osborne et al., 2010), have not yet percolated into the field of wound healing angiogenesis research. Discrete and continuum approaches to modeling each come with their own advantages and disadvantages: while discrete models can provide a level of detail that continuum models cannot, they are not amenable to most mathematical analytic techniques in the way that continuum models typically are. One could argue that the physical and biological phenomena that constitute wound healing could more realistically be captured using a detailed discrete model than a continuum one. However, at a useful degree of complexity, the simulation of each interaction within a healing wound is computationally demanding and approaches involving multiple regimes might need to be considered (Flekkøy et al., 2001; Flegg et al., 2012b, 2015; Franz et al., 2013). The question of which model framework one ought to utilize should ultimately come down to the resolution of the available experimental data and the motivating research question. As the clinical and experimental observations incorporated into most previous models of wound healing have been largely at the macroscopic level (for example, measurements of wound area Byrne et al., 2000), the continuum framework has been a reasonable choice in these cases.

With the trends in increasing computing power, better predictions from computational models of wound healing angiogenesis can be expected. Firstly, continuum reaction-transport models can be solved with fine resolution in 2D and 3D, with complex wound geometries motivated by clinical data. Continuum mathematical models, informed by relevant clinical and biological data, can then be used to make predictions on the treatment of wounds with novel therapies, thus reducing the need for expensive and time-consuming laboratory experiments and clinical trials. Secondly, detailed discrete cellular simulations will become within reach. With the data from individual cell imaging and tracking experiments, the behavior of individual cells can be captured in discrete mathematical models. Discrete cellular models will provide a quantitative framework to test hypotheses on the biochemical and biomechanical mechanisms that control cell behavior during wound healing angiogenesis.

Models of wound healing and wound healing angiogenesis should, ideally, be developed in close collaboration with clinicians and/or biologists to ensure the models capture reality accurately enough to be able to answer the overarching research question. This requires a common language of communication between collaborating mathematicians and biologists. With this review, we hope to draw attention to the wide array of possibilities that an increased cross-disciplinary dialogue could facilitate.

### Conflict of interest statement

The authors declare that the research was conducted in the absence of any commercial or financial relationships that could be construed as a potential conflict of interest.
